# Ectopic Expression of *GsPPCK3* and *SCMRP* in *Medicago sativa* Enhances Plant Alkaline Stress Tolerance and Methionine Content

**DOI:** 10.1371/journal.pone.0089578

**Published:** 2014-02-25

**Authors:** Mingzhe Sun, Xiaoli Sun, Yang Zhao, Chaoyue Zhao, Huizi DuanMu, Yang Yu, Wei Ji, Yanming Zhu

**Affiliations:** Key Laboratory of Agricultural Biological Functional Gene, Northeast Agricultural University, Harbin, P.R. China; Louisiana State University and A & M College, United States of America

## Abstract

So far, it has been suggested that phosphoenolpyruvate carboxylases (PEPCs) and PEPC kinases (PPCKs) fulfill several important non-photosynthetic functions. However, the biological functions of soybean PPCKs, especially in alkali stress response, are not yet well known. In previous studies, we constructed a *Glycine soja* transcriptional profile, and identified three PPCK genes (*GsPPCK1*, *GsPPCK2* and *GsPPCK3*) as potential alkali stress responsive genes. In this study, we confirmed the induced expression of *GsPPCK3* under alkali stress and investigated its tissue expression specificity by using quantitative real-time PCR analysis. Then we ectopically expressed *GsPPCK3* in *Medicago sativa* and found that *GsPPCK3* overexpression improved plant alkali tolerance, as evidenced by lower levels of relative ion leakage and MDA content and higher levels of chlorophyll content and root activity. In this respect, we further co-transformed the *GsPPCK3* and *SCMRP* genes into alfalfa, and demonstrated the increased alkali tolerance of *GsPPCK3*-*SCMRP* transgenic lines. Further investigation revealed that *GsPPCK3*-*SCMRP* co-overexpression promoted the PEPC activity, net photosynthetic rate and citric acid content of transgenic alfalfa under alkali stress. Moreover, we also observed the up-regulated expression of *PEPC*, *CS* (citrate synthase), *H^+^-ATPase* and *NADP-ME* genes in *GsPPCK3*-*SCMRP* transgenic alfalfa under alkali stress. As expected, we demonstrated that *GsPPCK3*-*SCMRP* transgenic lines displayed higher methionine content than wild type alfalfa. Taken together, results presented in this study supported the positive role of *GsPPCK3* in plant response to alkali stress, and provided an effective way to simultaneously improve plant alkaline tolerance and methionine content, at least in legume crops.

## Introduction

As a versatile crop, alfalfa (*Medicago sativa* L.) is used for pasture, hay, silage and green-chop, and acts in crop rotation through its positive effects on soil fertility and soil structure [1]. Due to its versatility, high productivity, high feed value and potential roles in soil improvement and soil conservation, alfalfa is grown over a wide range of climatic conditions [1], [2]. However, environmental challenges, especially soil salinity and alkalinity, not only seriously restrict alfalfa yield, but also affect nodules formation and symbiotic nitrogen-fixation capacity [3]. With the global climate change and the global shrinkage of arable lands, a grimmer reality of soil salinity and alkalinity is painted, hence more and more attentions have been paid to exploring alfalfa cultivation on marginal lands.

As reported earlier, of the 831 million hectares salt-alkali soils on the world, saline and alkaline soils underline 397 (47%) and 434 (53%) million hectares, respectively [4], [5]. What’s worse, in northeast China, over 70% of land area is alkaline grassland [6]. Alkaline soil is characterized by high pH, high exchangeable sodium, poor fertility, dispersed physical properties and low water content [7]. Recently, a handful of researches suggested that, compared with salt stress, alkaline stress always caused much stronger inhibition of plant growth and development [8]. Unfortunately, until now, little attention has been paid on the molecular mechanisms of plant adaptation to alkaline stress.

Phosphoenolpyruvate carboxylase (PEPC; EC4.1.1.31) is a kind of tightly controlled cytosolic enzyme which functions in carbon fixation during photosynthesis [9]–[11]. PEPC kinase (PPCK) controls the phosphorylation state and bioactivity of PEPCs. In recent years, PPCK genes have been identified in different higher plants, including two for Arabidopsis [12], three for rice [13], and four for soybean [14]. Recently, several lines of direct evidence supported that PEPCs and PPCKs fulfilled important non-photosynthetic functions, particularly in plant response to environmental challenges. One of the best studied examples was that salt stress remarkably increased PPCK activity [15]–[17]. Further investigation revealed that salt stress not only increased PPCK gene expression levels but also decreased PPCK protein degradation rates [17]. Moreover, PEPC/PPCK activity in Arabidopsis and poplar was also regulated by alkali stress [12], [18].

Besides high stress tolerance, another important desired trait for alfalfa is high nutritional value in terms of essential amino acids. As we know, legume plants are deficient in the sulfur-containing amino acids, namely, methionine and cysteine [19]. Methionine is nutritionally essential for mammals, but at low levels in legume. Unlike plants, mammals could not synthesize methionine; hence they have to obtain it from their diets. In this respect, methionine deficiency obviously limited the nutritional value of legumes; therefore, increasing the methionine content has become another important goal for legumes breeding. To solve this problem, in a previous study, we designed and synthesized the *SCMRP* gene according to the maize methionine-rich 10 ku zein protein [20], [21]. A similar study reported that overexpression of the maize 10 ku zein gene increased the sulphur-containing amino acids of transgenic potato [22].

For these reasons, in this study, we aimed to generate the transgenic alfalfa not only with higher alkali tolerance but also with higher methionine content. Based on the *Glycine soja* (G07256) microarray data, we isolated and characterized an alkali stress responsive gene *GsPPCK3*. Our results demonstrated that *GsPPCK3* overexpression in alfalfa improved plant tolerance to alkali stress. In this case, we co-transformed the *GsPPCK3* and *SCMRP* genes into alfalfa, and demonstrated that the transgenic alfalfa displayed not only higher alkali tolerance but also higher methionine content. Taken together, results presented in this study demonstrated the biological function of *GsPPCK3* under alkali stress, and provided an effective way to simultaneously improve plant alkaline tolerance and methionine content, at least in legume crops.

## Results

### Isolation and Sequence Analysis of the *GsPPCK3* Gene

**Figure 1:**
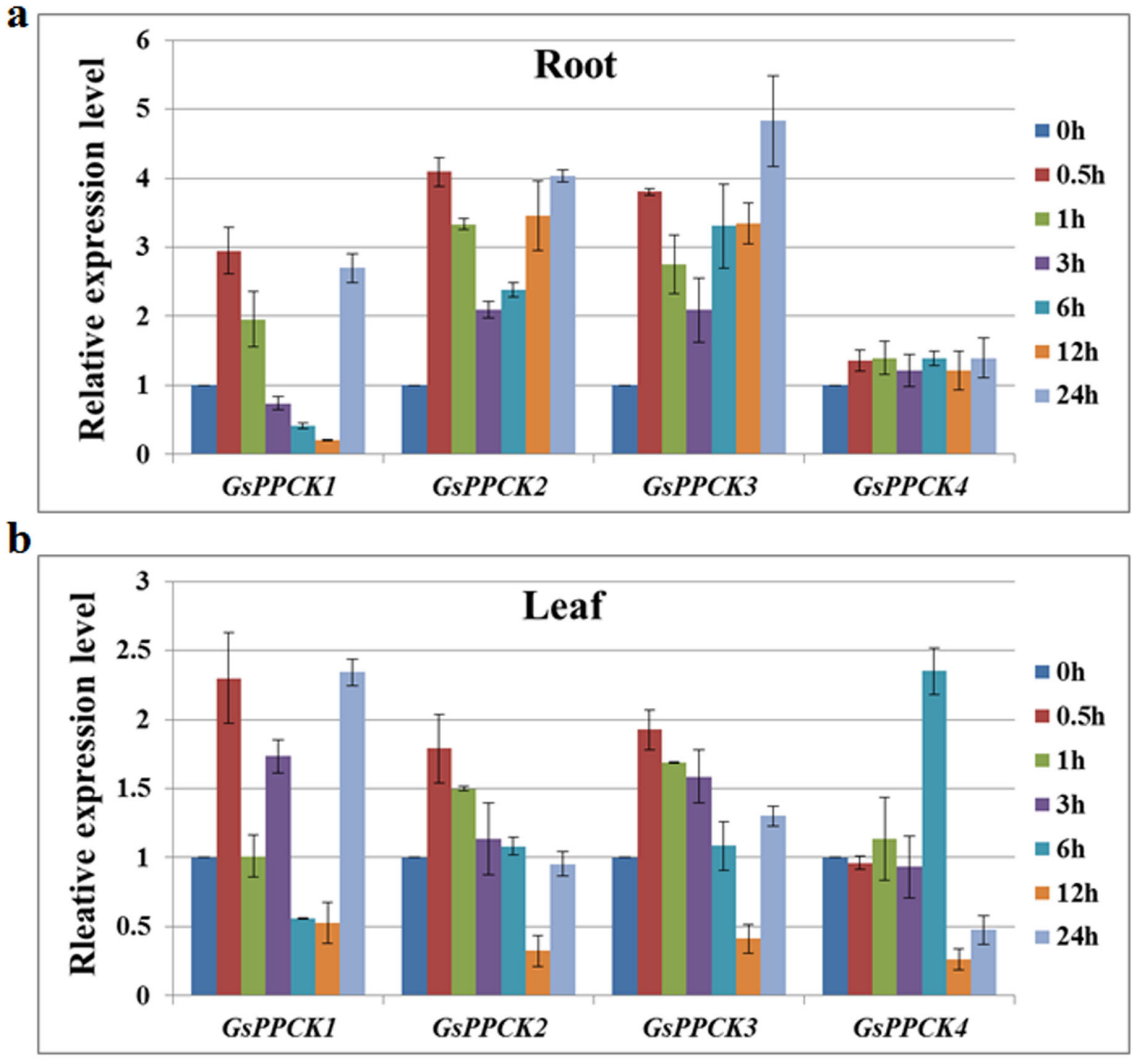
Expression patterns of the *Glycine soja* PPCK family genes under 50 mM NaHCO_3_ (pH 8.5) treatment based on the microarray data. a. Expression patterns of the PPCK family genes under alkali stress in *Glycine soja* roots. b. Expression patterns of the PPCK family genes under alkali stress in *Glycine soja* leaves.

In a previous study, we constructed a transcriptional profile of *Glycine soja* (G07256) roots and leaves in response to salt-alkali stress (50 mM NaHCO_3_, pH 8.5), by using the Affymetrix® Soybean Genome Array [23]. Three PEPC kinase genes *GsPPCK1*, *GsPPCK2* and *GsPPCK3* were identified as potential stress responsive genes (Fig. 1). Contrarily, the last PPCK gene *GsPPCK4* essentially did not respond to salt-alkali stress, only with an increase at 6 h in leaves.

In this study, we obtained the full length coding region of *GsPPCK3* by using homologous cloning strategy. *GsPPCK3* contained a complete open reading frame (ORF) of 915 bp encoding 304 amino acids with an estimated molecular weight (Mr) of 34 000 and a theoretical pI of 5.35. A BLASTP search at NCBI showed that GsPPCK3 shared 90%, 84%, 69% and 61% sequence identity with the *Glycine max* GmPPCK3 (GeneBank Accession: NP_001238645), GmPPCK2 (NP_001236660), GmPPCK1 (NP_001241581) and GmPPCK4 (NP_001237016), respectively. It is worth noting that GsPPCK3 contained a 30 amino acid length sequence (KLLLASVFLFDIIFGGFCVDGIFGCFVFVG) at its C-terminus, which did not exist in any soybean and Arabidopsis PPCKs. By comparing the DNA and mRNA sequences of *GsPPCK3* and *GmPPCK3*, we found that this 30 aa sequence was encoded by the 90 bp length intron of *GmPPCK3*.

**Figure 2:**
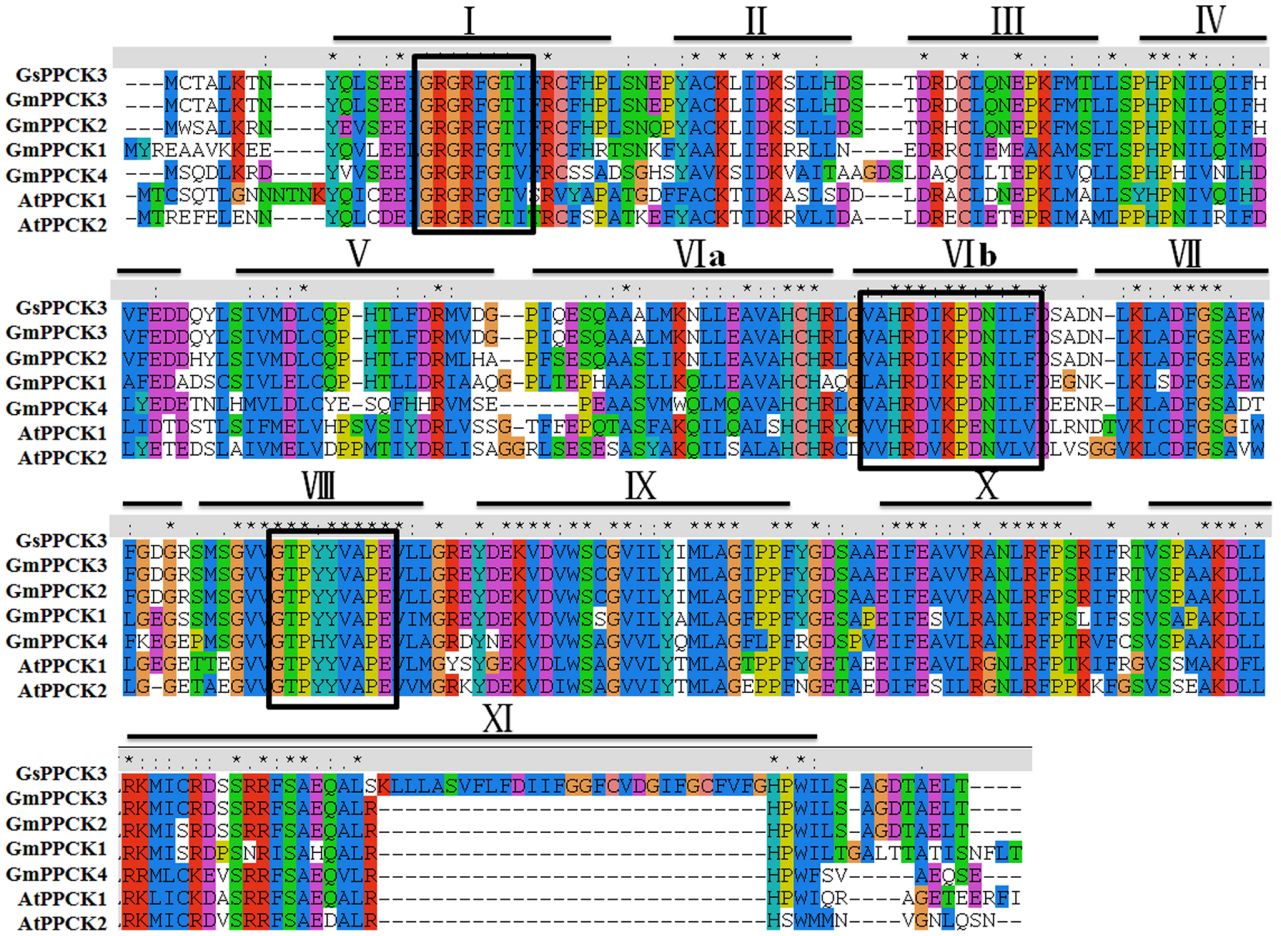
Multiple alignment between GsPPCK3 and homologous PPCKs from Arabidopsis and soybean based on the full-length amino acid sequences. The 11 subdomains of the catalytic domain were marked as solid lines. The protein kinase ATP-binding signature (GxGxxG), the Ser/Thr kinase active site signature (VAHRDIKPDNILF) and the conserved G-T/S-XX-Y/F-X-APE motif were marked as black solid boxes. Sequences were aligned by using ClustalW, and gaps were introduced to maximize alignment.

In order to get better understanding of GsPPCK3 structure, we compared the amino acid sequences of GsPPCK3 with PPCK homologs from *Glycine max* and *Arabidopsis thaliana* (AtPPCK1 and AtPPCK2) (Fig. 2). Protein sequence analysis revealed that GsPPCK3 comprised a minimal Ser/Thr kinase domain, closely related to the catalytic domain of plant calcium-dependent protein kinases (CDPKs) without the N- and C-terminal extensions. GsPPCK3 contained all of the 11 conserved subdomains required for kinase activity [24], [25] (Fig. 2), along with a protein kinase ATP-binding signature (GxGxxG, residues 16–21) and a Ser/Thr kinase active site signature (VAHRDIKPDNILF, residues 128–140) [26]. Similar to other PPCKs, GsPPCK3 also contained a conserved G-T/S-XX-Y/F-X-APE motif in subdomain VIII, indicating that GsPPCK3 was a potential Ser/Thr kinase rather than a Tyr kinase [27]. Second structure prediction revealed that GsPPCK3 contained two transmembrane domains at the C-terminus (residues 189–207, and residues 263–289), implying the potential membrane localization.

### Expression Patterns of *GsPPCK3* in Response to Alkaline Stress and in Different Tissues

**Figure 3:**
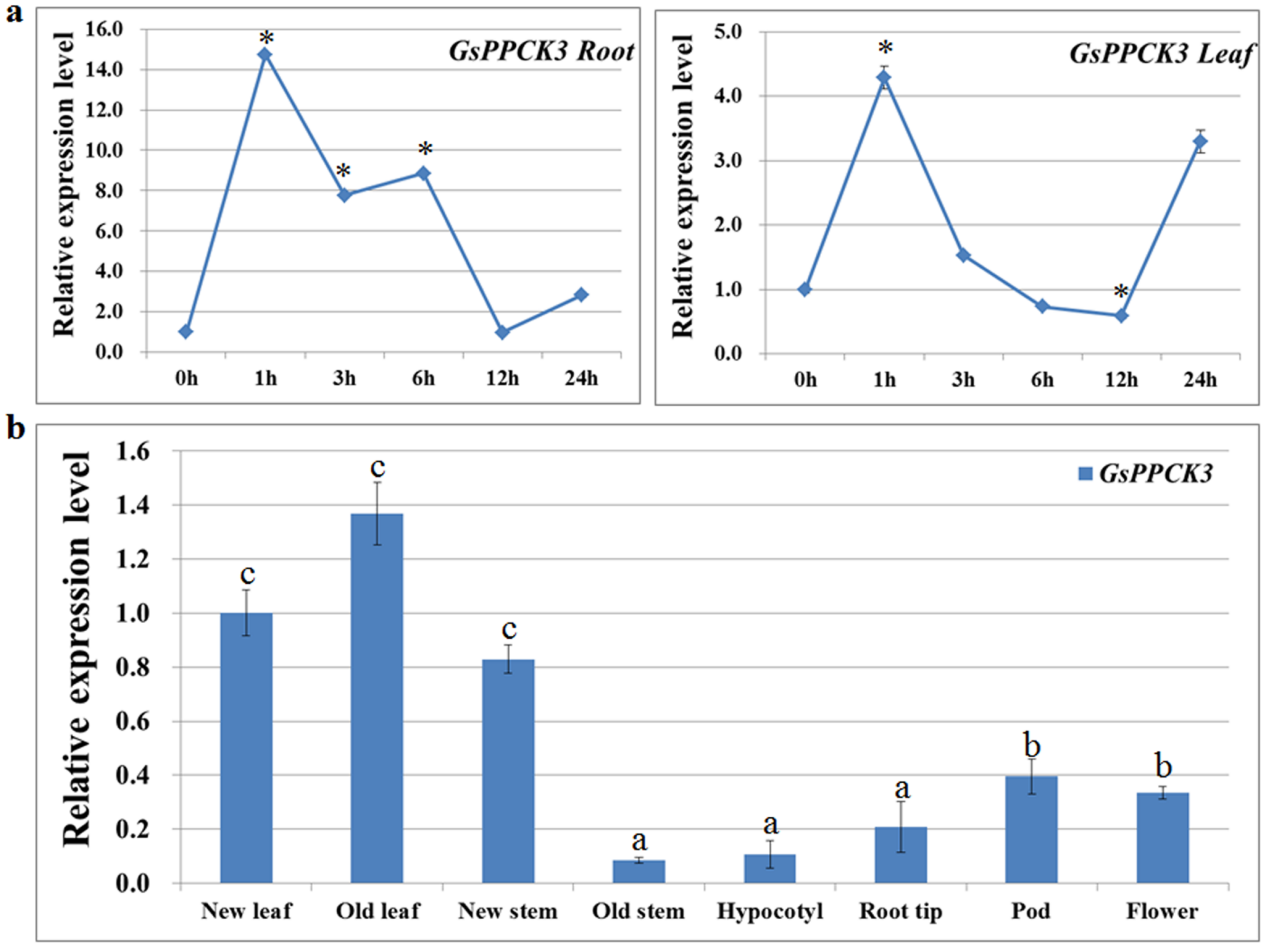
Expression patterns of *GsPPCK3* in *Glycine soja*. a. Expression levels of *GsPPCK3* were up-regulated by alkali stress in both roots and leaves. Total RNA was extracted from leaves and roots of the 3-week-old *Glycine soja* seedlings treated with 50 mM NaHCO_3_ (pH 8.5) for the indicated time points, respectively. Relative transcript levels were determined by quantitative real-time PCR analysis with *GAPDH* as an internal control. The mean values from three fully independent biological repeats and three technical repeats were shown. *P<0.05; **P<0.01 by Student’s t-test. b. Tissue expression specificity of *GsPPCK3* in *Glycine soja*. Total RNA was extracted from different tissues of *Glycine soja* seedlings. Significant differences (P<0.05 by Duncan’s Multiple Range Test) were indicated by different lowercase letters.

In an attempt to verify the induced expression of *GsPPCK3* under alkali stress, we carried out the quantitative real-time PCR analyses. Consistent with the microarray data, *GsPPCK3* expression in both leaves and roots of *Glycine soja* seedlings was greatly and rapidly induced by alkali stress (Fig. 3A). After 50 mM NaHCO_3_ (pH8.5) treatment, *GsPPCK3* displayed an obvious increase and reached a maximum point at 1 h. It is noteworthy that the induction degree of *GsPPCK3* in roots was significantly higher than that in leaves. The possible reasons for the stronger response might be that plant roots were the exact sites of perception and injury for stresses, or there were different response mechanisms for *GsPPCK3* expression between roots and leaves. Anyway, it is obvious that *GsPPCK3* expression was greatly induced by alkali stress, suggesting an important role of *GsPPCK3* in plant response to alkali stress.

In order to get better understanding of *GsPPCK3* expression patterns, we further investigated the spatial specific expression of *GsPPCK3* in *Glycine soja* seedlings (Fig. 3B). The real-time PCR results showed that, among the eight tissues detected in this study, *GsPPCK3* displayed the highest expression level in leaves, which could be explained by the important role of *GsPPCK3* in photosynthesis. Contrarily, the *GsPPCK3* transcript level in roots was relatively lower, even though it exhibited a greater degree of alkali stress induction in roots. This difference led us to propose the possibility of different mechanisms and different roles of *GsPPCK3* between roots and leaves in plant response to alkali stress.

### *GsPPCK3* Overexpression Confers Enhanced Alkaline Tolerance in Transgenic Alfalfa

**Figure 4:**
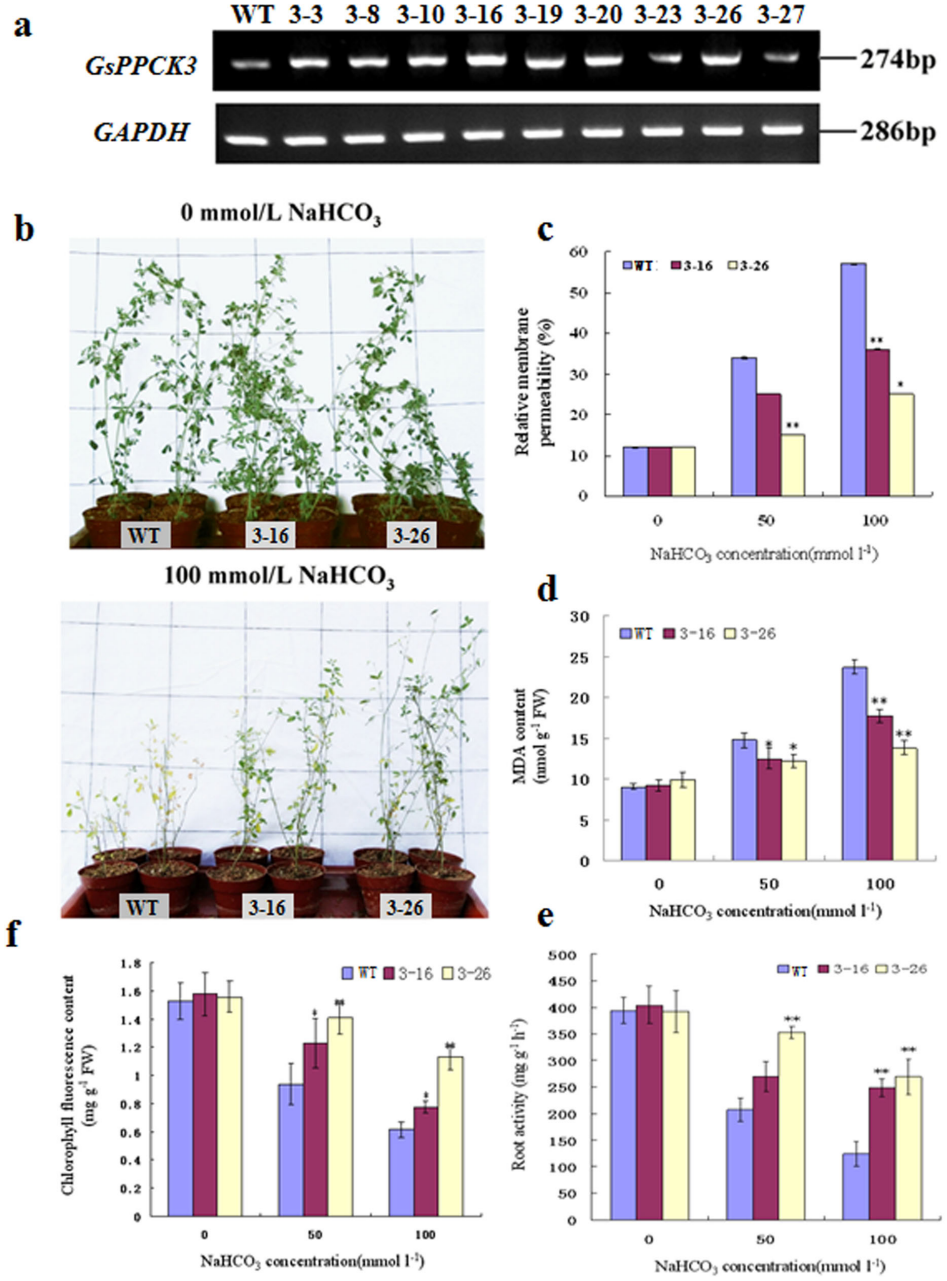
*GsPPCK3* overexpression in alfalfa conferred enhanced alkaline tolerance. **a.** Semi-quantitative RT-PCR identification of *GsPPCK3* transgenic alfalfa. **b.** Growth performance of WT and transgenic lines under control conditions or 100 mM NaHCO_3_ treatment. Photographs were taken 12 days after initial treatment. **c.** The relative membrane permeability of WT and transgenic plants. **d.** The MDA content of WT and transgenic plants. **e.** The root activity of WT and transgenic plants. **f.** The total chlorophyll content of WT and transgenic plants. For phenotypic analysis of *GsPPCK3* transgenic alfalfa, the 3-week-old WT and *GsPPCK3* transgenic plants with similar sizes were treated with 1/8 Hoagland nutrient solution containing either 0, or 50, or 100 mM NaHCO_3_ every 3 days for a total of 12 days.

The stress induced expression of *GsPPCK3* gave us an insight into its potential role in response to alkali stress. To precisely investigate the biological and physiological function of *GsPPCK3* under alkaline stress, we transformed *GsPPCK3* into the wild type (WT) *M. Sativa* through the *Agrobacterium tumefaciens*-mediated transformation strategy (Figure S1A). By using PCR and semi-quantitative RT-PCR analyses, we identified a total of nine transgenic lines (Fig. 4A), and two of them, with relatively higher expression levels (3–16 and 3–26), were selected to examine the response to alkaline stress.

Under control conditions, transgenic lines showed no obvious differences in growth performance compared with WT. After 50 or 100 mM NaHCO_3_ treatment for 14 d, both the WT and transgenic lines showed growth retardation in a dose-dependent manner. However, the growth inhibition of transgenic lines was less severe than that of WT (Fig. 4B). In details, WT plants exhibited severe chlorosis and even death, whereas the transgenic lines maintained continuous growth after 100 mM NaHCO_3_ treatment.

We further compared the relative membrane permeability (Fig. 4C), MDA content (Fig. 4D), chlorophyll content (Fig. 4E) and root activity (Fig. 4F) of WT and *GsPPCK3* transgenic lines, respectively. No obvious differences were observed between WT and transgenic lines under control condition. As expected, alkali stress significantly increased the relative membrane permeability and MDA content, but decreased the total chlorohpyll content and root activity of both WT and transgenic plants. However, the transgenic lines showed relatively lower levels of electrolyte leakage and MDA content (Fig. 4C, D), but higher levels of total chlorohpyll content and root activity than WT (Fig. 4E, F) (*P<0.05; **P<0.01 by Student’s t-test). Taken together, these results strongly suggested that *GsPPCK3* overexpression improved the alkali tolerance of transgenic alfalfa.

### Increased Alkaline Tolerance of *GsPPCK3-SCMRP* Overexpression Transgenic Alfalfa

**Figure 5:**
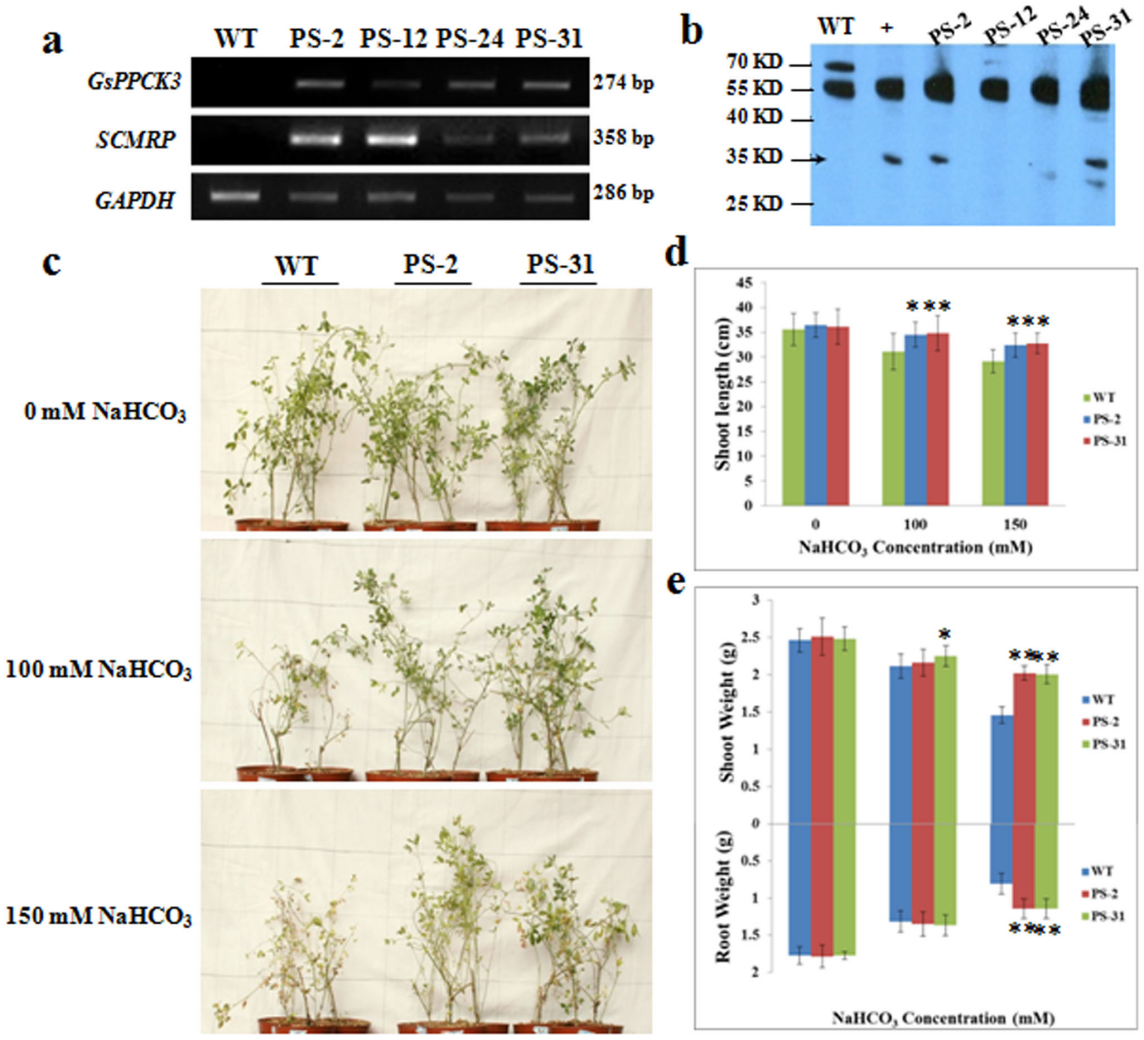
Increased alkaline tolerance of *GsPPCK3-SCMRP* overexpression transgenic alfalfa. **a.** Semi-quantitative RT-PCR analysis of *GsPPCK3* and *SCMRP* expression levels in WT and trangenic lines. **b.** Western blot identification of *GsPPCK3-SCMRP* transgenic alfalfa. **c.** Growth performance of WT and transgenic lines after alkali treatment. Photographs were taken 15 days after initial treatment. **d.** Shoot length of the WT and transgenic lines. **e.** Shoot weight and root weight of the WT and transgenic lines. For phenotypic analysis of *GsPPCK3-SCMRP* transgenic alfalfa, the 4-week-old plants were treated with 1/8 Hoagland nutrient solution containing either 0, or 100, or 150 mM NaHCO_3_ every 3 days for a total of 15 days.

Considering the deficiency in sulfur-containing amino acids, we then co-transformed the *GsPPCK3* and *SCMRP* genes into *M. Sativa* (Fig. S1B), in an attempt to obtain the transgenic alfalfa with not only higher stress tolerance but also higher methionine content. We identified a total of four transgenic lines by using PCR and semi-quantitative RT-PCR assays (Fig. 5A). Based on western blot analysis, two transgenic lines (PS-2 and PS-31) were selected for further studies (Fig. 5B).

We firstly compared the alkali tolerance between WT and *GsPPCK3*-*SCMRP* transgenic alfalfa. As shown in Fig. 5C, alkali stress obviously inhibited the growth of both WT and transgenic plants; however, transgenic lines displayed significantly taller (Fig. 5D) and more biomass accumulation than WT (Fig. 5E). Specifically, in the presence of 150 mM NaHCO_3_, the shoot length was 29.14 cm for WT, 32.31 cm for PS-2 and 32.72 cm for PS-31, and the shoot/root weight was 1.46/0.81 g for WT, 1.91/1.08 g for PS-2 and 1.9/1.08 g for PS-31, respectively (*P<0.05; **P<0.01 by Student’s t-test). These results suggested that *GsPPCK3* and *SCMRP* co-transformation promoted seedling growth of the transgenic alfalfa under alkali stress.

### Improvement of PEPC Activity, Photosynthetic Rate and Citric Acid Content in Transgenic Alfalfa

**Figure 6:**
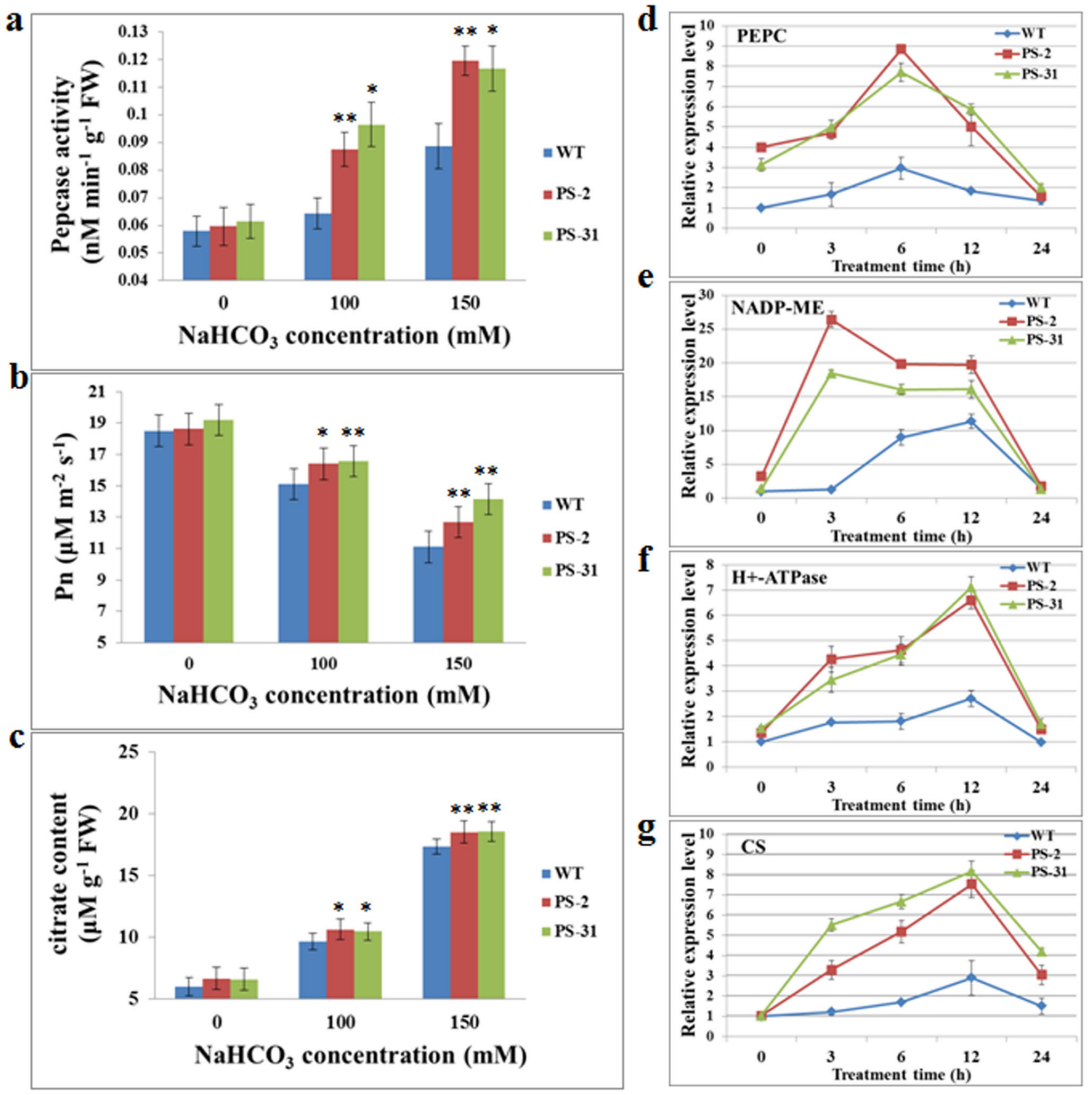
*GsPPCK3* overexpression altered a set of physiological indices and expression levels of stress responsive genes. **a.** The PEPC activity of the WT and *GsPPCK3-SCMRP* transgenic lines under alkali treatment. **b.** The net photosynthetic rate of the WT and *GsPPCK3-SCMRP* transgenic lines. **c.** The citric acid content of the WT and transgenic lines. **d.** Increased expression levels of *PEPC* in *GsPPCK3-SCMRP* transgenic plants under alkali stress (50 mM NaHCO_3_, pH 8.5). **e.** Increased expression levels of *NADP-ME* in *GsPPCK3-SCMRP* transgenic plants. **f.** Increased expression levels of *H^+^-ATPase* in *GsPPCK3-SCMRP* transgenic plants. **g.** Increased expression levels of *CS* in *GsPPCK3-SCMRP* transgenic plants. To explore expression patterns of stress-responsive genes, the 4-week-old WT and *GsPPCK3-SCMRP* transgenic seedlings after shoot cottage were treated with 50 mM NaHCO_3_ (pH 8.5) for 0, 3, 6, 12 and 24 h, respectively. Relative transcript levels were determined by quantitative real-time PCR with *GAPDH* as internal reference, and were normalized to WT plants at 0 h. Values represented the means of three fully independent biological replicates, and three technological replicates for each.

To further elucidate the influence of *GsPPCK3* overexpression in alfalfa, PEPC activity, which was regulated by PPCK phosphorylation [28], [29], was determined by measuring the seedling crude extracts. As shown in Fig. 6A, alkaline stress improved the PEPC activity in both WT and transgenic lines, however, an obvious up-regulation of PEPC activity was observed in the transgenic lines. Specifically speaking, PEPC activity of the transgenic lines was 31.64% (PS-2) and 34.78% (PS-31) higher than that of WT, respectively (*P<0.05; **P<0.01 by Student’s t-test).

It has been suggested that photosynthetic rate decreased proportionally, along with the increase of NaHCO_3_ concentration [30]. Considering the function of PEPC/PPCK in photosynthesis, we measured the net photosynthesis (Pn) rates of both WT and transgenic lines under alkali stress. The quantitative analysis revealed that Pn of transgenic alfalfa was significantly higher than that of WT, in the presence of 100 or 150 mM NaHCO_3_ (Fig. 6B) (*P<0.05; **P<0.01 by Student’s t-test). These results suggested that overexpression of *GsPPCK3* led to an obvious increase of Pn under alkali stress, maybe by activating PEPCs.

Citric acid content was considered to be an indicator of plant response to pH challenge resulting from alkaline stress [31]. As expected, plants accumulated more citric acids under alkali stress than under control conditions. Compared with WT, the transgenic lines exhibited significantly higher levels of citric acid content, after 100 or 150 mM NaHCO_3_ treatment (Fig. 6C) (*P<0.05; **P<0.01 by Student’s t-test). Collectively, these results demonstrated that the alleviation of high pH damage caused by alkali stress in transgenic alfalfa might be related to the elevated levels of PEPC activity, photosynthetic rate and citric acid content, due to *GsPPKC3* overexpression.

### *GsPPCK3* Overexpression Up-regulated the Expression Levels of Several Stress Responsive Genes

Previous studies showed that H^+^-ATPase and NADP-ME maintained the homeostasis of the cytosolic pH value [32], [33]. In view of the increased PEPC activity and citric acid content of transgenic alfalfa, we examined the expression patterns of *PEPC*, *CS* (Citrate synthase), *H^+^-ATPase* and *NADP-ME* after 100 mM NaHCO_3_ treatment. The real-time PCR analysis indicated that expression of *PEPC*, *CS*, *H^+^-ATPase* and *NADP-ME* was induced by alkali stress in both WT and transgenic lines. Expectedly, the expression levels in transgenic plants were significantly higher than that in WT (Fig. 6D-G), which explained the up-regulation of the PEPC activity and citrate acid content. These results implied that *GsPPCK3* overexpression promoted the accumulation of transcript expression levels of the stress responsive genes, which might be helpful for the intracellular pH regulation under alkali stress.

### Increased Methionine Content in Leaves of *GsPPCK3-SCMRP* Transgenic Alfalfa

In addition to the enhanced alkali tolerance, we also determined the content of 16 amino acids in leaves of both WT and transgenic plants, to verify if *GsPPCK3*-*SCMRP* co-transformation increased the methionine content (Table 1). As expected, *GsPPCK3-SCMRP* transgenic lines displayed significantly higher levels of methionine content than WT (*P<0.05; **P<0.01 by Student’s t-test), without obvious changes for other amino acids. Specifically speaking, the methionine contents were 0.97±0.04% in WT plants, 2.27±0.89% (2.34 folds to WT) in transgenic line PS-2 and 2.23±0.67% (2.29 folds) in line PS-31. Taken together, these results suggested that *GsPPCK3-SCMRP* co-overexpression not only enhanced the alkaline tolerance, but also increased the methionine content of transgenic alfalfa.

**Table 1:** Amino acids content of the WT and *GsPPCK3-SCMRP* transgenic alfalfa.

Amino acid	WT	Content (%) PS-2	PS-31
Asp	6.91±0.39	6.84±0.32	7.38±0.51
Thr	6.48±0.00	6.27±0.06	6.40±0.06
Ser	3.65±0.05	3.58±0.01	3.68±0.10
Glu	8.74±0.09	8.69±0.01	8.76±0.11
Gly	19.28±0.20	17.53±0.37	18.76±0.83
Ala	9.40±0.07	9.28±0.02	9.38±0.27
Cys	3.88±0.05	3.99±0.08	3.77±0.09
**Met**	**0.97±0.04**	**2.27±0.89** **	**2.23±0.67** *
Ile	4.16±0.10	4.17±0.14	3.96±0.06
Leu	8.38±0.10	8.49±0.18	8.13±0.24
Tyr	1.53±0.27	1.38±0.28	1.14±0.19
Lys	6.98±0.10	7.18±0.16	6.75±0.06
NH3	1.62±0.16	1.80±0.14	1.82±0.29
His	3.49±0.09	3.55±0.16	3.58±0.07
Arg	4.96±0.03	5.00±0.07	4.86±0.17
Pro	9.51±0.01	9.98±0.08	9.41±0.24

*P<0.05;

**P<0.01 by Student’s t-test.

## Discussion

Saline-alkaline stress, as a kind of widespread environmental stress with significantly negative impact on plant growth, severely reduces crop productivity and affects agricultural production worldwide. It has been suggested that alkaline soil, characterized by high NaHCO_3_/Na_2_CO_3_ content, caused injury to plants not only through salt stress, but also through alkali stress [34]–[36]. Plants could homeostatically maintain the intracellular pH value and ion concentration in a temperate range [32], [37]–[39]. Unfortunately, up to now, most studies emphasized on salt stress [40]–[43], and no slightly definite mechanism was proposed about plant response to alkaline stress.

*Glycine soja*, the wild ancestor of cultivated soybean (*Glycine max*), could normally germinate and grow in the alkaline soil with a pH value at 9.02 [44]. In previous studies, we constructed the global transcriptional profile of *Glycine soja* (G07256) under alkali stress (50 mM NaHCO_3_, pH 8.5), and three of the four PPCK genes *GsPPCK1*, *GsPPCK2* and *GsPPCK3* were identified as putative stress responsive genes (Fig. 1) [23]. Contrarily, *GsPPCK4* essentially did not respond to alkali stress, only with an increase at 6 h in leaves. Consistent with previous researches, these results further supported the evolutionary relationship of soybean PPCKs [14], [45]. *PPCK1*, *PPCK2* and *PPCK3* shared a high similarity and belonged to the same legume PPCK subfamily, while *PPCK4* represented a high divergence, outlier to the legume PPCK.

In a previous study, we isolated and characterized one of the alkali stress responsive PPCK genes *GsPPCK1*, and found that overexpression of *GsPPCK1* in alfalfa significantly improved plant tolerance to alkali stress [46]. In the present study, we focused on the expression pattern and biological function of *GsPPCK3*. We cloned the full length *GsPPCK3*, and found that it shared 90%, 84%, 69% and 61% sequence identity with *GmPPCK3*, *GmPPCK2*, *GmPPCK1* and *GmPPCK4*, respectively. Protein sequence analysis revealed a unique motif consisting of 30 amino acids in subdomain XI of GsPPCK3, which was not found in any soybean and Arabidopsis PPCKs. This unique sequence was encoded by the 90 bp length intron of *GmPPCK3*, indicating the existence of different transcripts in *Glycine soja*. Furthermore, GsPPCK3 protein contained all conserved subdomains required for kinase activity [24], [25] (Fig. 2), including a protein kinase ATP-binding signature, a Ser/Thr kinase active site signature [26] and a conserved G-T/S-XX-Y/F-X-APE motif, as well as two transmembrane domains.

*GsPPCK3* expression was greatly and rapidly induced by alkali stress in both leaves and roots (Fig. 3A). Previous researches also suggested the induced expression of PPCKs under environmental stress. For example, salt stress significantly increased the transcript accumulation of *SbPPCK1* and *SbPPCK2* in sorghum [17]. Moreover, *GsPPCK1* was also induced by alkali stress and positively regulated the alkali tolerance of transgenic alfalfa [46]. Therefore, the alkali induced expression indicated an important role of *GsPPCK3* in plant response to alkali stress. It is worth noting that the stress induction degree of *GsPPCK3* in roots was significantly higher than that in leaves. It is possible that plant roots were the exact sites of perception and injury for stresses, or there were different response mechanisms for *GsPPCK3* expression between roots and leaves.

It has been reported that PPCK genes showed spatial expression specificity. For example, *OsPPCK1* and *OsPPCK3* displayed obviously high expression levels in roots [13], while *GmPPCK2* and *GmPPCK3* showed high transcription levels in root nodules [14]. Except for root nodules, *GmPPCK3* also showed relatively higher expression levels in stems, flowers and young leaves. In this study, we found that *GsPPCK3* displayed the highest level in leaves but relatively lower in roots (Fig. 3B). As we know, PEPC undergoes an important function in carbon fixation during photosynthesis, and its activity was largely regulated by PPCK phosphorylation [28], [29]. It is reasonable to speculate that the high levels of *GsPPCK3* transcripts in leaves could make sure of the high PEPC activity and thereby the effective photosynthesis of plant leaves. Considering the difference in the transcript levels and alkali induction degrees of *GsPPCK3* between roots and leaves, one could speculate that in leaves, most *GsPPCK3* products were used for carbon fixation in photosynthesis process, but in roots, *GsPPCK3* mainly functioned in alkali response.

To further confirm the biological function of *GsPPCK3* in alkali response, we transformed *GsPPCK3* into alfalfa and carried out the alkali tolerance assays. We gave four lines of direct evidence showing the increased tolerance and possible mechanisms of *GsPPCK3* transgenic alfalfa in response to alkali stress (Fig. 4, 5). Firstly, *GsPPCK3* overexpression significantly promoted plant growth under alkali stress. *GsPPCK3* transgenic lines displayed much better at plant height, shoot weight and root weight than WT under alkali stress (Fig. 5D, E). Secondly, *GsPPCK3* overexpression alleviated the damage caused by alkali stress, as evidenced by an obvious decrease in the relative ion leakage (Fig. 4C) and MDA content (Fig. 4D), but an increase in root activity (Fig. 4E) [37], [47]–[49]. Thirdly, *GsPPCK3* overexpression in alfalfa resulted in increased PEPC activity (Fig. 6A), increased chlorophyll content (Fig. 4F) and thereby the increased Pn rate (Fig. 6B), which in turn could promote plant growth under alkali stress. We also observed the higher expression levels of *PEPC* gene in transgenic alfalfa (Fig. 6D). In this context, we proposed the hypothesis that *GsPPCK3* regulated the PEPC activity not only through protein phosphorylation process, but also through gene transcription regulation. Finally, overexpression of *GsPPCK3* led to more effective response to high pH stress by increasing the citrate acid content (Fig. 6C) [50]. The up-regulation of *CS* gene expression (Fig. 6G) in transgenic plants explained the increase of citrate acid content [51], [52]. Meanwhile, we also suggested relatively higher expression level of *H^+^-ATPase* in transgenic lines under alkali stress (Fig. 6F), which was also helpful for intracellular pH regulation. Based on these results, we speculated that *GsPPCK3* regulated the gene expression and enzyme activity involved in photosynthesis and pH regulation, alleviated damage caused by alkali stress, and finally promoted plant growth under alkali stress.

On the other hand, we also obtained the transgenic alfalfa with not only higher alkali tolerance but also higher methionine content, by co-transforming the *GsPPCK3* and *SCMRP* genes. As a kind of nutritionally essential amino acid, methionine was found to be at low level in legume. In this study, we significantly improved the methionine content of transgenic alfalfa by ectopically expressing the *SCMRP* gene, which was designed and synthesized according to the maize methionine-rich 10 ku zein protein [20], [21]. Similarly, it has been reported that overexpression of the maize 10 ku zein gene in potato could increase the contents of sulphur-containing amino acids [22]. During the past decades, a series of efforts have been made to increase the methionine content by using genetic engineering methods, for example altering expression levels of the methionine-rich storage proteins [53]–[55], or increasing the soluble content of methionine [56]–[60].

Collectively, here we provided an effective way to simultaneously improve plant alkaline tolerance and methionine content, at least in legume crops. For the first time, we gave exact evidence for a PPCK protein from *Glycine soja*, and provided insights into a plausible mechanism by which *GsPPCK3* positively controlled plant tolerance to alkali stress.

## Materials and Methods

### Plant Material and Growth Conditions

*Glycine soja* (G07256) seeds, obtained from Jilin Academy of Agricultural Sciences (Changchun, China), were treated with 98% sulfuric acid for 10 min, washed five times with sterile water, and then kept in complete darkness with humidity for 2–3 days to promote germination. The seedlings were transferred and grown in 1/4 Hoagland solution for 3 weeks at 24–26°C and 16 h light/8 h dark cycles. To explore the expression profiles of *GsPPCK3* under alkali stress, the 3-week-old seedlings were treated with 1/4 Hoagland solution containing 50 mM NaHCO_3_ (pH 8.5) for 0, 1, 3, 6, 12 and 24 h, respectively. Equal amounts of leaves and roots were harvested and stored at −80°C after snap-frozen in liquid nitrogen.

*Medicago Sativa* L. was kindly obtained from Heilongjiang Academy of Agricultural Sciences (Haerbin, China), and grown in green house under controlled environmental conditions (24–26°C, 16 h light/8 h dark cycles, 600 µmol m^−2^ s^−1^, 80±5% relative humidity). To investigate the expression patterns of stress-responsive genes, the 4-week-old seedlings after shoot cottage were treated with 1/4 Hoagland solution containing 50 mM NaHCO_3_ (pH 8.5) for 0, 3, 6, 12 and 24 h, respectively. Samples were harvested and stored as described above.

### Isolation and Sequence Analysis of the *GsPPCK3* Gene

Total RNA was extracted from the 3-week-old *Glycine soja* seedlings, by using RNeasy Plant Mini Kit (Qiagen, Valencia, CA, USA), and subjected to cDNA synthesis by using SuperScript™ III Reverse Transcriptase kit (Invitrogen, Carlsbad, CA, USA). The full-length coding region of *GsPPCK3* was PCR amplified with gene specific primers (5′-AAGATAGAATGTGCACAGCCCTAAAG-3′ and 5′-TTCTCAAGTGAGTTCAGCCGTGTC-3′), and cloned into pGEM-T vector (Promega, Madison, WI, USA) for sequencing.

Sequence similarity was examined by using the on-line BLASTP program at NCBI (http://blast.ncbi.nlm.nih.gov/Blast.cgi). Homology searches were executed by BLASTP at Phytozome (http://www.phytozome.net/soybean). TMHMM (http://www.cbs.dtu.dk/services/TMHMM/) and Tmpred (http://www.ch.embnet.org/software/TMPRED_form.html) were used to predict the transmembrane domains.

### Quantitative Real-time PCR Analyses

Total RNA extraction and cDNA synthesis were operated as described above. Quantitative real-time PCR analyses were performed by using SYBR Premix ExTaq™ II Mix (TaKaRa, Shiga, Japan) on an ABI 7500 sequence detection system (Applied Biosystems, Carlsbad, CA, USA). The glyceraldehyde-3-phosphate dehydrogenase genes *in G. soja* (Accession: DQ355800) and *M. sativa* (Accession: Medtr3g085850) were used as internal references, respectively. Expression levels for all candidate genes were calculated by using the 2^-ΔΔCT^ method, and the relative intensities were normalized as described previously [61]. To enable statistical analysis, three fully independent biological replicates and three technical repeats were conducted. Primers used for quantitative real-time PCR were designed using Primer 5 software and listed in Table 2.

**Table 2:** Gene-specific primers used for quantitative real-time PCR assays.

Gene name	Gene ID	Primer Sequence (5′ to 3′)	PCR productsize (bp)
*GsGAPDH*	DQ355800	Forward: GACTGGTATGGCATTCCGTGTReverse: GCCCTCTGATTCCTCCTTGA	121
*GsPPCK3*		Forward: CGCAGAACAAGCCTTGAGTAAGReverse: CCACCACGAGTAGACCACCTT	264
*MtGAPDH*	Medtr3g085850	Forward: GTGGTGCCAAGAAGGTTGTTATReverse: CTGGGAATGATGTTGAAGGAAG	286
*PEPC*	L39371.2	Forward: CATTGGCTCGGTTGTTCTCCReverse: TCTGTGCGACTTTGATGAGGTC	159
*H^+^-ATPase*	AJ132891	Forward: GGCAGCCCTCTACCTACAAGTCReverse: AGCAATCATAAAAGCACCCAAT	121
*NADP-ME*http://www.ncbi.nlm.nih.gov/nucleotide/357520876?report = genbank&log$ = nucltop&blast_rank = 1&RID = 35MT7XMC01R	XM_003630679.1	Forward: TAGGTGGAGTTCGTCCTTCAGCReverse: AGGTCATAGTATTCCTTCCCAGTTG	133
*Citrate Synthase*	HM030734.1	Forward: TCTATATGGACCTCTTCATGGTGGReverse: TGAGCTTTCGTTTCCTGGCT	122

### Generation of Transgenic Alfalfa

In order to investigate the influence of *GsPPCK3* and *SCMRP* on plant stress tolerance and methionine content, we constructed the expression vectors carrying the *GsPPCK3* gene alone (Fig. S1A), and the *GsPPCK3* and *SCMRP* genes together (Fig. S1B), respectively. The *GsPPCK3* gene was inserted to the bone vector pBEOM (made in our lab) under the control of the cauliflower mosaic virus (CaMV) 35S promoter, with the binding enhancers E12 and omega. The *SCMRP* gene was under the control of double CaMV35S promoter and omega sequence. The *Bar* gene was used as the selectable marker. The recombinant vectors were introduced into *A. tumefaciens* strain EHA105, and then transformed into *M. sativa* by using the cotyledonary node method. The transformants were selected by using 0.5 mg L^−1^ glufosinate ammonium, and regenerated shoots were rooted on 1/2 Murashige and Skoog (MS) medium. At last, the glufosinate-positive seedlings were transplanted into soil and grown in green house under controlled conditions.

Presence of the *GsPPCK3* and *SCMRP* genes in the glufosinate-positive plants was confirmed by PCR analysis using CaMV35S promoter specific forward primer and *Bar* gene specific reverse primer (5′-CCTGTGCCTCCAGGGAC-3′ and 5′-GCGGTCTGCACCATCGTC-3′). *GsPPCK3* transcript levels in the PCR-positive plants were analyzed by semi-quantitative RT-PCR analysis using gene specific primers (5′-CCCTCCTTTCACCTCACC-3′ and 5′-GAACCGAAGTCCGCCAGT-3′). Expression levels of the *SCMRP* gene were examined by using a pair of specific primers (5′-CAGCAGGGTCTCGCTTCACT-3′ and 5′-GCAGATTCCAATGCCACAAT-3′). The alfalfa *GAPDH* gene was used as an internal control. And the PCR- and RT-PCR positive seedlings were subjected to western blot analysis with specific polyclonal antibody to the C-terminus of GsPPCK3 protein (CHPWILSAGDTAELT).

### Phenotypic Analysis of Transgenic Alfalfa Under Alkali Stress

The lignified WT and transgenic alfalfa plants were propagated by stem cuttings, and about 2 weeks later, adventitious roots appeared. The seedlings were transplanted into plastic culture pots filled with a mixture of peat moss: soil (1∶1; v/v), irrigated with 1/8 Hoagland nutrient solution and grown in green house under controlled conditions. For phenotypic analysis of *GsPPCK3* transgenic alfalfa, the 3-week-old WT and transgenic plants with similar sizes were exposed to alkali stress by irrigating with 1/8 Hoagland solution containing either 0, or 50, or 100 mM NaHCO_3_ every 3 days for a total of 12 days. For phenotypic analysis of *GsPPCK3-SCMRP* transgenic alfalfa, the 4-week-old plants were exposed to alkali stress by irrigating with 1/8 Hoagland solution containing either 0, or 100, or 150 mM NaHCO_3_ every 3 days for 15 days.

The total chlorophyll content was determined in 80% (v/v) acetone extract according to the method of Arnon [62]. The relative electrolyte leakage was measured using a conductivity meter (DDSJ-308A, Shanghai, China) as described previously [63]. The malon dialdehyde (MDA) content was determined according to the protocol described by Peever et al. [64]. The biological activity of roots from alkali-treated WT and transgenic plants was assessed by measuring root dehydrogenase activity using triphenyltetrazolium chloride (TTC) reduction technique as described [65]. The citric acid content was determined by using a spectrophotometer (UV-2550, Shimadzu, Japan) at the absorbance of 490 nm, according to the method of Zhu [66].

The net photosynthesis rate was determined by using the LI-6400 chamber (LI-COR Biosciences, Lincoln, NE, USA). During the experiments, the light intensity was 500 µmol photons m^−2^ s^−1^, and the CO_2_ concentration was in the range of 350 to 400 µmol mol^−1^. The flow rate was adjusted to 500 µmol s^−1^, and the leaf temperature was maintained at 25°C. For statistical analysis, three independent experiments were conducted with at least 5 independent plants per genotype and experiment.

PEPC activity was measured by the coupled spectrophotometric method at 340 nm and 30°C as previously described by Gonzalez [67]. Total proteins of the WT and OX alfalfa leaves were extracted by using the 0.1 M Tris-HCl (pH 7.5) solution, containing 20% (v/v) glycerol, 1 mM EDTA, 10 mM MgCl_2_, 10 µg mL^−1^ chymostatin, 10 µg mL^−1^ bestatin, 10 µg mL^−1^ leupeptin, 1 mM PMSF, 1 µg mL^−1^ microcystin-L/R (L and R are two variable amino acids in the structure of microcystin), and 14 mM β-mercaptoethanol. Protein concentration was determined according to the method of Bradford (1976) [68]. All of the above numerical data were subjected to statistical analyses using EXCEL 2007 and/or IBM SPSS statistics 19, and analyzed by Student’s T-test and/or Duncan’s Multiple Range Test.

### Determination of Amino Acid Content

Leaves of WT and transgenic alfalfa were fixated at 105°C for 15 min, and then dried to constant weight at 80°C. About 100 mg dried leaves were crushed, suspended in 6 M HCl, and then hydrolyzed at 110°C for 22 h with nitrogen replacement. The hydrolysate was dried under reduced pressure, dissolved in 0.02 M HCl, and subjected to the amino acid analyzer (Hitachi, Japan).

## Supporting Information

Figure S1 Schematic representation of the constructs for Agrobacterium tumefaciens-mediated transformation into Medicago sativa. a, Schematic representation of the GsPPCK3 overexpression construct. b, Schematic representation of the GsPPCK3-SCMRP overexpression construct.
(TIF)
