# Supplementary figures and images for: Ectopic Expression of GsPPCK3 and SCMRP in Medicago sativa Enhances Plant Alkaline Stress Tolerance and Methionine Content
Source: PLoS One. 2014 Feb 25;9(2):e89578. doi: 10.1371/journal.pone.0089578 (PMC3934933; doi:10.1371/journal.pone.0089578)

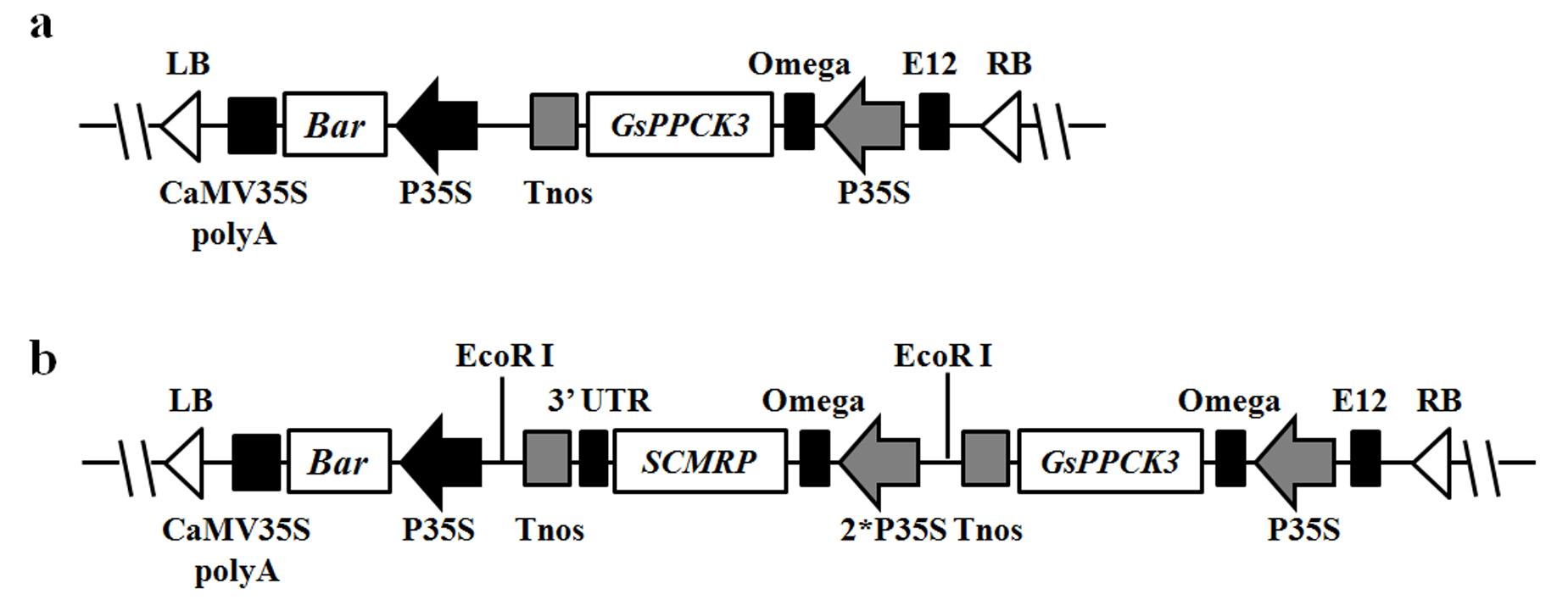

Supplement: Figure S1 — Schematic representation of the constructs for Agrobacterium tumefaciens-mediated transformation into Medicago sativa. a, Schematic representation of the GsPPCK3 overexpression construct. b, Schematic representation of the GsPPCK3-SCMRP overexpression construct. (TIF) [file pone.0089578.s001.tif]
